# Astrovirology and terrestrial life survival

**DOI:** 10.6026/973206300200146

**Published:** 2024-02-29

**Authors:** Paul Shapshak, Milad Zandi, Charurut Somboonwit, John T. Sinnott

**Affiliations:** 1 Department of Internal Medicine, Morsani College of Medicine, University of South Florida, Tampa, Florida 33606, USA; 2 Hepatitis Research Center, Department of Virology, Faculty of Medicine, Lorestan University of Medical Sciences, Khorramabad, IRAN

**Keywords:** Astrovirology, astrobiology, exoplanets, exomoons, solar system, asteroids, near Earth Ryugu, comets, atmosphere, temperature, elements, metallicity, pre-biotic chemistry, tholin, biochemistry, molecular biology, goldilocks, signature, complexity, chaos theory, catastrophe theory, singularity, attractor, biosecurity, biodefense, NIH, CDC, WHO

## Abstract

Microbial organisms have been implicated in several mass extinction events throughout Earth's planetary history. Concurrently, it can be reasoned from recent viral pandemics that viruses likely exacerbated the decline of life during these periods of mass extinction. The fields of exovirology and exobiology have evolved significantly since the 20th century, with early investigations into the varied atmospheric compositions of exoplanets revealing complex interactions between metallic and non-metallic elements. This diversity in exoplanetary and stellar environments suggests that life could manifest in forms previously unanticipated by earlier, more simplistic models of the 20th century. Non-linear theories of complexity, catastrophe, and chaos (CCC) will be important in understanding the dynamics and evolution of viruses.

## Abbreviations:

NIH = National Institutes of Health

CDC = Centers for Disease Control

WHO = World Health Organization

## Note:

Goldilocks: Goldilocks conditions include extreme and non-extreme terrestrial environments where life exists.

## Astrovirology and Astrobiology: expanding the framework:

Stedman *et al.* provided a comprehensive review of astrovirology and Schwieterman *et al.* thoroughly investigated exoplanetary biosignatures indicative of life [1-2]. Building upon their work, we explore additional environmental and microbiological parameters that could influence the emergence and evolution of life, across extraterrestrial environments. By drawing parallels with terrestrial events, we hypothesize similar or divergent outcomes on exoplanets under analogous or unique conditions. The application of complexity, catastrophe, and chaos (CCC) theories is central for modeling non-linear, conditional scenarios that offer a more nuanced understanding than simply linear models.

## factorial exo-planet research:

The Perryman exoplanet handbook emerges as a critical resource, challenging the oversimplified goldilocks hypothesis through its exhaustive compilation of exoplanetary research. This data-driven book forecloses the inadequacy of single-factor theories for the origins of life and in explaining habitability of exoplanets. Advancements in astronomy and chemistry highlight the necessity for further technological innovation. Also, theoretical sophistication allows us to deepen our understanding of possible exoplanetary events. However, caution is advised against drawing definitive conclusions, as anomalies necessitate ongoing reassessment and refinement. The advent of quantum computing and neural networks is revolutionizing both our field as well as invigorating the search for extraterrestrial life by facilitating computational analyses [3,4, 5].

Limiting the problem to Earth-bound molecular biology and biochemistry, because it is terrestrial (and the only life ever detected), neglects the wider panoramas needed to evaluate the origin of life elsewhere, without missing it. Given the single occurrence of life as seen on the Earth, there have been cataclysmic events. At least five en mass extinction events occurred during Earth's planetary history. Are such events anticipated for life discovered elsewhere? Will extinction events be analogous among divergent life forms and their disparate biochemical and environmental conditions? Moreover, will such extinction events be engendered while terrestrial life is propagated throughout the solar system? An important corollary question is when does extra-terrestrial intelligence (ETI) develop in an exobiological history? On Earth, intelligence (defined scientifically) developed among mammals, presumably serendipitously, after five mass extinctions [3-6].

## The five terrestrial mass extinctions are briefly listed as follows:

[1] Approximately 440 million years ago: Ordovician period in the Paleozoic Era where 85% life is destroyed due to continental drift and climate change. Increased plant evolution and biodiversity considered contributory [7].

[2] Approximately 375 million years ago: Devonian period in the Paleozoic Era where 80% of life is destroyed due to lower ocean oxygen content, quick air temperature cooling, and possibly volcanic eruptions and meteor impacts. Increased plant evolution and biodiversity are also considered contributory [7].

[3] Approximately 250 million years ago: Permian period in the Paleozoic Era where 96% life destroyed due to asteroid strikes, volcanic activity, and climate change. In addition, it is important to note the involvement of microbial life upheaval and spread. Microbial life not only exploded but may have also contributed to the mass extinctions. A huge amount of methane release may have been causative because via natural genetic engineering steps, Archaea (Methanosarcina) received two acetate metabolizing genes, transferred from bacteria. [8,9,10].

[4] Approximately 200 million years ago: End of Triassic period in the Mesozoic Era where 50% life destroyed due to volcanic activity, molten basalt flooding, global climate change, ocean levels and pH change.

[5] Approximately 65 million years ago: End of the Cretaceous period in the Mesozoic Era where 75% life destroyed due to asteroid/meteor impact [11].

It should be noted that durations between extinctions are 65, 75, 50, 135, and 65 million years. Additionally, note that the probability of survival across the entire duration, since the beginning of terrestrial life is 0.15 x 0.20 x 0.04 x 0.50 x 0.25 = 0.00015. The survival chance of cellular life since the beginning is 0.015%. However, many factors are missing from this simple calculation, including the change from RNA to DNA life at the beginning, the influence of viruses and other microbial forms of life, and the effects exerted by evolutionary changes in biochemistry and molecular biology. Thus, during the extinctions, viruses, bacteria, and fungi, may have increasingly infected animals and plants, as their health deteriorated. (Paradoxically, viruses are currently the most numerous life forms on the Earth. [2]) Complexity, chaos, and catastrophe (CCC) theory studies are germane and needed [12-13]. Sustained and increased complexity may lead to catastrophe-chaos points, due to non-Darwinian 'forces', yet to be identified. These CCC points would be analogous to mathematical singularities and attractor-centers.

## Unraveling intra-solar and extra-solar system life:

Intra-solar system and extra-solar system observations are multifactorial and mentioned briefly as follows. Since the Earth is the only planet, thus far, where life was detected, it is of interest to compare planets within our solar system and exoplanets. There are several exoplanet and asterosystem archives including TESS, Kepler, K2, KELT, and UKIRT databases [14, 15]. These online libraries cross-correlate and collate information and astronomical data on exoplanets and their host stars. Tools are provided to analyze data. For example, Perryman reported that among 494 exoplanets, 106 were detected through transit methods in 105 systems. These findings list characteristics such as the planets' names, detection methods, stellar magnitudes, distances, masses, radii, orbital periods, semi-major axes, eccentricities, and stellar parameters alongside transit references [3,15]. Furthermore, the mass composition of our solar system includes: total mass of the planets 446.6M⊕, total mass of satellite/moons 0.104M⊕, total mass of asteroids 0.0003M⊕, and total mass of meteors and comets 10-9 M⊕. (M⊕ is the mass of the Earth) [3, 16]. It should be noted that the combined masses of the asteroids, meteors, and comets are less than 10-5% mass of the solar system. This finding reduces the probability that viable organisms could have been carried among the planets in our solar systems, let alone among stars and their extra-solar systems. It has been hypothesized, though, that protosolar systems already contain organic precursors. Moreover, it also means that negligible water and biocompounds could have been transferred at any significant level. That should reduce the chances of panspermia as a means of dispersal of life and its antecedents among stars and their planets [4,17,18, 19,20].

## Molecular signatures:

The molecular signatures of our own solar system present interesting but limited data for analysis. Venus showcases a predominance of atmospheric carbon dioxide, Earth has a nitrogen and oxygen-rich atmosphere, Mars has carbon dioxide air, and the gas giants have hydrogen and helium compositions. Diversity is apparent and these variances, especially the contrast between the silicate and iron cores of terrestrial planets and the gas giants' hydrogen and helium atmospheres, suggest a broad spectrum of conditions under which the emergence of life, as we understand it, could occur. Conversely, though, unlike current conditions, life appeared on Earth under reducing not oxidizing atmospheric conditions [3,20].

The exploration for life beyond Earth extends into challenging propositions, includes the potential habitability of Europa and Io, with their theorized subsurface oceans heated by tidal forces and Jupiter's magnetospheric dynamics. The super-Earth GJ1214, with its elevated temperature of 450 oK, joins a list of candidates where life's biochemical signatures might be detectable, hinting at a universe teeming with possibilities. Chemicals in exoplanet atmospheres considered to be life-related include methane, oxygen, ozone, nitric oxide, carbon dioxide, methyl-mercaptan, methyl chloride, and of course, water. The quest to identify life's precursors has been long-standing, since the 19th century, yet challenges remain, particularly in distinguishing genuine prebiotic molecules from human-interaction contaminants on meteorites. The advent of ultrasensitive analytical technologies has underscored the necessity for immaculate sample collection and analysis conditions, as demonstrated by Oba, Yakano, and their team's pioneering work on the Ryugu asteroid samples. Their discovery of over 20,000 compounds, including amino acids in unexpectedly low abundance compared to other meteorites, marks a significant surge in prebiotic chemistry research, suggesting a complex biochemical evolution, not yet understood. [3,4,15, 20,21,22]

## Metallicity, volatility, exoplanet atmosphere temperatures:

The realm of metallicity and potential life forms underscores the vastness of conditions under which life might exist. We previously addressed the issue of metallicity and diverse possible forms of extraterrestrial life. Perryman's insights into the properties of metals and other elements, based on their condensation temperatures, offer a nuanced view of the cosmos's chemical diversity. From ultra-refractory to highly volatile elements, the classification paves the way for envisaging life forms, radically different from those on Earth. Moreover, the temperature ranges actually observed in exoplanet atmospheres, reaching up to several thousand degrees Kelvin, further complicate the search for life. The detection of aluminum oxide in the atmosphere of WASP-43b, for instance, demonstrates the variety of chemical environments that must be considered in the search for extra-terrestrial life. This diversity, coupled with the evolving capabilities of available analytical methods, suggests many future discoveries, as the depths of space are probed for signs of life beyond our solar system. [3,23, 24].

Specifically, Perryman's Exoplanet handbook summarizes properties of several metal types and other elements as follow, based on 50% condensate temperatures at 10 Pascals (Pa). Refractory and volatile elements: condensation temperatures give the volatility groupings for the elements. These results are:

[1] Ultra-refractory: Temperature >1650 oK: the metals Os, Re, W; the lithophiles Al, Hf, Sc, Th, Y, Zr; and the heavy rare earth elements Gd, Tb, Dy, Ho, Er, Tm, Lu.

[2] Highly refractory: Temperature 1650-1500 oK: the metals Ir, Mo, Ru; the lithophiles Ca, Nb, Ta, Ti, U; and the light rare earth elements La, Pr, Nd, Sm. rock-forming elements: Ca, Al, Ti, Fe, Si, Mg.

[3] Refractory, 1500-1360 oK: the metals Pt, Rh; the lithophiles Ba, Be, Ce, Sr, V, Yb.

[4] The common elements: Temperature 1360-1290 oK: Mg, Si, Fe, Co, Cr, Ni, Pd; and the lithophile Eu.

[5] Moderately volatile: Temperature 1290-704 oK: the siderophiles Ag, As, Au, Bi, Cu, Ga, Ge, P, Pb, Sb, Te; lithophiles Cs, B, K, Li, Na, Mn, Rb, Zn; the halogens Cl, F.

[6] Volatile: Temperature 704-371 oK: the chalcophiles Cd, In, S, Se, Tl; the siderophile Sn; the halogens Br, I.

[7] Highly volatile: Temperature < 371 oK: C, N, O; noble gases; Hg; and the more abundant organic compounds of H, C, N, O.

Among this plethora of conditions, many different life forms might develop that would be utterly divergent from terrestrial life. The actual temperature ranges of exoplanets are relevant in concert with these descriptions of metallicity and volatility. Among a plethora of observations of atmospheric metals, for example, Chubb and colleagues identified aluminium oxide (AlO) in the WASP-43b atmosphere, which is 835oK (For comparison, as an example of a setpoint limit, Al transitions to free ionic Al+ at 3,000 oK) [3, 23,24].

## Biodefense:

Terrestrial viruses, in addition to the recent SARS pandemic, include many extreme untreatable pathogens, termed risk group-4 (RG-4) viruses. Biodefensive measures that are taken against such viruses should also include terrestrial microorganisms that evolve into pathogens, off-world. Interestingly and along these lines, there is a clear danger of spreading terrestrial-derived micro-organisms via space-probes with or without astronauts. In addition, tholins are found throughout the solar system and have been demonstrated to be nutritional substrates for microorganisms in terrestrial laboratories. As a post-script, Biodefense must be extended to life or life-products that include metallicity [23,25, 26,27,28]. A pervasive hazard is that terrestrial-derived microbes as well as extra-terrestrial microbiota may utilize tholins as an energy source substrate, thus providing a means for serendipitous spread.

## Conclusion:

We briefly examine several key issues relating to exovirology, exobiology, and the search for the origin of life, including exoplanet atmospheres, a variety of extinction events on Earth, and several characteristics of the elements. Much work needs to be done to relate their significance for the origin of life. Nonetheless, they illustrate the diversity of conditions that exist on exoplanets. Due to the exigencies of randomness, extreme conditions, and CCC theory (for non-linear analysis), there is considerable room for additional work and this approach benefits from continued technological advancement. Whatever may be life's antecedents, more than one molecular premise range should be anticipated. Moreover, rigorous statistics should be engaged, in which dissimilar types of probabilities stated under diverse conditions and for unrelated types of life need to be accomplished. In fine, continued increased complexity may lead to catastrophic-chaotic singularities and attractor-centers.

## References

[1] Schwieterman EW (2018). Astrobiology..

[2] Berliner A (2018). Astrobiology..

[3] Perryman M (2018). The Exoplanet Handbook.

[4] Chyba CF, Sagan C (1992). Nature..

[5] Rivera-Valentin EG (2021). Astrobiol..

[6] https://psycnet.apa.org/record/2011-12794-017.

[7] Hull P, Darroch SA (2013). Ecosystem Paleobiology and Geobiology..

[8] Rothman DH (2014). PNAS (USA)..

[9] Wald C (2014). Nature..

[10] https://www.newscientist.com/article/mg19726421-900-mass-extinctions-the-microbes-strike-back/.

[11] https://www.thoughtco.com/the-5-major-mass-extinctions-4018102?print.

[12] Day R, Letts GK (1999). JSocPolThought..

[13] Viswanath D, Sahutoglu S (2010). SIAM Review..

[14] https://exoplanetarchive.ipac.caltech.edu.

[15] https://exoplanet.eu/home/.

[16] http://www.exoplanet.eu/catalog/.

[17] https://en.m.wikipedia.org/wiki/Panspermia.

[18] https://www.sciencedirect.com/science/article/abs/pii/B9780323957175000098.

[19] Wickremasinghe C (2011). Int J Astrobiol..

[20] Miller SL (1953). Science..

[21] Seager S, Demming D (2010). Ann. Rev. Astron and Astroph..

[22] Oba Y (2023). Nature Communications..

[23] Shapshak P (2021). Bioinformation..

[24] Chubb KL (2020). Astr and Astroph.

[25] https://www.cdc.gov/labs/pdf/SF__19_308133-A_BMBL6_00-BOOK-WEB-final-3.pdf.

[26] Sinnott JT (2023). Bioinformation..

[27] Sinnott JT (2023). Bioinformation..

[28] Stoker CR (1990). Icarus..

